# Improved Bacteriostatic and Anticorrosion Effects of Polycaprolactone/Chitosan Coated Magnesium via Incorporation of Zinc Oxide

**DOI:** 10.3390/ma14081930

**Published:** 2021-04-12

**Authors:** Hamid Reza Bakhsheshi-Rad, Esah Hamzah, Wong See Ying, Mahmood Razzaghi, Safian Sharif, Ahmad Fauzi Ismail, Filippo Berto

**Affiliations:** 1 Advanced Materials Research Center, Department of Materials Engineering, Najafabad Branch, Islamic Azad University, Najafabad, Iran; mahmood.razzaghi@gmail.com; 2 Faculty of Engineering, Universiti Teknologi Malaysia, Johor Bahru, Johor 81310, Malaysia; seeying1202@gmail.com (W.S.Y.); safian@utm.my (S.S.); 3 Advanced Membrane Technology Research Center (AMTEC), Universiti Teknologi Malaysia, Johor Bahru, Johor 81310, Malaysia; afauzi@utm.my; 4 Department of Mechanical and Industrial Engineering, Norwegian University of Science and Technology, 7491 Trondheim, Norway

**Keywords:** magnesium, PCL/CS/ZnO coating, corrosion resistance, antibacterial activity biocompatibility

## Abstract

Magnesium has been recognized as a groundbreaking biodegradable biomaterial for implant applications, but its use is limited because it degrades too quickly in physiological solutions. This paper describes the research on the influence of polycaprolactone (PCL)/chitosan (CS)/zinc oxide (ZnO) composite coating (PCL/CS/ZnO) on the corrosion resistance and antibacterial activity of magnesium. The PCL/CS film presented a porous structure with thickness of about 40–50 μm, while after incorporation of ZnO into the PCL/CS, a homogenous film without pores and defects was attained. The ZnO embedded in PCL/CS enhanced corrosion resistance by preventing corrosive ions diffusion in the magnesium substrate. The corrosion, antibacterial, and cell interaction mechanism of the PCL/CS/ZnO composite coating is discussed in this study. In vitro cell culture revealed that the PCL/CS coating with low loaded ZnO significantly improved cytocompatibility, but coatings with high loaded ZnO were able to induce some cytotoxicity osteoblastic cells. It was also found that enhanced antibacterial activity of the PCL/CS/ZnO coating against both Escherichia coli (*E. coli*) and Staphylococcus aureus (*S. aureus*) bacteria, while less significant antibacterial activity was detected for uncoated Mg and PCL/CS coating. Based on the results, the PCL/CS coatings loaded with low ZnO content may be recommended as a candidate material for biodegradable Mg-based orthopedic implant applications.

## 1. Introduction

Over the past few years, biomaterials are mainly from stainless steels, titanium, and Co–Cr alloys. However, these metals release toxic ions into body fluid on degradation [1]. Besides, there is a significant difference in the mechanical properties of these metal alloys with actual bone tissue [2]. This may result in implant failure due to the difference in the elastic modulus which has become one of the main concern among researchers, as this difference in elastic modulus results in a higher occurrence possibility of stress-shielding [3,4]. Stress distribution changes as the implanted parts tend to receive a higher load than the real bones; the real bones remodel and reconstruct to a more porous or thinner structure as it is less stimulated by load [5]. To address this issue, magnesium (Mg) alloys were introduced in the orthopedic and trauma surgery [6], since they can provide preferred characteristics of implantation, such as biocompatibility, biodegradability, and mechanical strength [7].

Mg density and its alloys (1.74 g/cm^3^–1.85 g/cm^3^) are highly comparable to that of human cortical bone (1.75 g/cm^3^). Mg is naturally found in bone tissue and is essential for human metabolism [8]. Mg^2+^ ions formed during biomaterial degradation also contribute to tissue healing and growth [9,10]. Thus, Mg and its alloy have become a good candidate to fabricate degradable bone implants, such as screws, bone plates, and pins [11]. Implants made of Mg and its alloys can eliminate the need for further surgery to remove the implant, as it will degrade when the damaged tissue grows back [12]. However, Mg corrodes too rapidly in vivo and produces a large amount of hydrogen gas beneath the skin, limiting their wide applications [13]. To deal with this problem, surface modification with PCL is implemented to decrease the initial degradation rate of biodegradable Mg, along with other biocompatible and antibacterial elements such as CS and zinc oxide, so that it will not corrode away before the bone tissue is entirely healed [14,15]. PCL is a non-toxic and biodegradable polyester prepared via ring-opening polymerization of the cyclic monomer ε-caprolactone and could be an effective barrier to inhibit the infiltration of the corrosive solution to Mg substrate and further protect the substrate [16]. However, PCL has insufficient surface wetting property, which is its hydrophobic property, leading to poor cell adhesion and proliferation [17]. Thus, PCL typically needs to be combined with other polymers such as CS to improve its cell adhesion property [15]. CS is a polysaccharide deacetylated from chitin and can be obtained from the exoskeletons of marine crustaceans, shellfish, and some fungi [18,19]. The CS properties, including biocompatibility in the human body, antioxidant activity, antimicrobial activity, hypoallergenic property, and anti-inflammatory activity, have attracted considerable attention in commercial applications [14,18]. In recent years, ZnO, particularly in the form of nanoparticles, has received attention as an antibacterial agent against bacterial infections. ZnO has proved to be a protectant that exhibits antiseptic actions. The study showed that ZnO thin film nanoparticles exhibit good antibacterial activity [1,7]. A study investigating ZnO antibacterial activity in synergy with other antibacterial agents found that a combination of ZnO with CS produced an outcome that showed better antimicrobial properties than CS alone [20,21,22]. Based on the literature, there is hardly any research done on the in-vitro characteristic of ZnO-incorporated PCL/CS coating, including antibacterial and biocompatibility performance. Hence, this study aims to evaluate the novel PCL/CS coating with different ZnO contents to improve the corrosion behavior and antibacterial performance of the substrate. It is hypothesized that the PCL/CS/ZnO coating significantly enhances the antibacterial and anticorrosion properties of Mg.

## 2. Materials and Experimental Procedure

### 2.1. Material Preparation and Characterization

**Figure 1:**
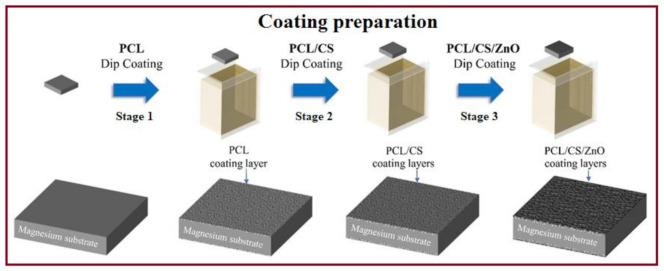
A schematic of the preparation of PCL/CS/ZnO-coated magnesium.

The fabrication process for providing the research samples and evaluations (Graphical Abstract) is schematically depicted in Figure 1. In this research, as-cast Mg substrates with dimensions of 15 × 10 × 10 mm^3^ were prepared according to Ref. [23]. The casting procedure of Mg is presented in the Supporting Information. An optical micrograph of as-cast pure Mg is depicted in Figure S1 (Supporting Information). For the coating process, PCL with a molecular weight (M_W_) of 80,000 g/mol, CS powder with Mw of 190−310 kDa (both purchased from Sigma-Aldrich, Gillingham, UK), and ZnO NPs, purchased from MyLab, were prepared. In this study, the coating was performed in three stages, which are comprehensibly presented in Supporting Information. For the first stage of coating, the PCL was measured and dissolved in 30 mL of dimethylformamide (DMF, 99.8% ACS, Sigma-Aldrich, Gillingham, UK). The solution was stirred for 2 h using a magnetic stirrer at 800 rpm. DMF was added until the total volume of the solution became 100 mL and was ready for dip coating. The mixture was then heated and stirred to form a homogeneous solution. The specimens were then immersed for 10 s in the suspension and dried for 5 min, and this process was repeated 5 times. For the second stage, 1.0 g of CS powder was added to the solution containing PCL, prepared the same way as the first stage, and stirred for 1 h at 800 rpm until a clear, viscous solution was obtained. The samples were then coated in the solution and dried similar to the first stage. For the final stage of the coating, after preparing the PCL/CS solution, an appropriate amount of xZnO, where *x* = 2, 4, and 6 wt.%, was added to the clear, viscous solution. Parameter such as the viscosity of the solution is affected with higher ZnO concentration which form droplets with larger pores as a result of an increase in the density of the coating solution droplets and their coalescences at higher concentration, as shown in Figure S2 (Supporting Information). The scanning electron microscope (SEM; JEOL JSM-6380LA, Tokyo, Japan) equipped with energy dispersive X-ray spectrometer (EDS; JEOL Ltd., Akishima, Japan) was employed to observe the surface morphology of uncoated and coated Mg before and after immersion. Fourier-transform infrared (FTIR; ALPHA-T, Bruker, Ettlingen, Germany) spectroscopy was used for detecting functional groups on the surface of coated specimens recorded in the spectral range of 4000 to 400 cm^−1^.

### 2.2. Bonding Strength and Wettability Characteristic

The bonding strength between the fibrous layer and the Mg substrate was evaluated according to ASTMF1044-05 standard using a universal tensile testing machine (RB 301 UNITECHM, Daejeon, Korea) at a rate of 5 mm/min. The wettability test was performed by employing a contact angle (CA) meter (GBX Digidrop, Romans-sur-Isère, France) to investigate the surface the change as a result of composite coating on Mg substrates. In this experiment, deionized water was used and carefully discharged onto the coated Mg substrates.

### 2.3. In Vitro Corrosion Behavior

All uncoated and coated specimens were immersed for 7 days in a cup containing 100 mL of simulated body fluid (SBF) at 37 °C temperature to investigate the corrosion behavior. The specimens were then removed from the cup, rinsed with distilled water, and dried at ambient conditions according to Ref. [24]. The uncoated and coated specimens were weighed before immersing the cup. After that, the in vitro corrosion rate (CR) (mm/year) was calculated employing the weight loss test based on the equation:

(1) CR=W/Atd

where W is the weight loss, A is the specimen area exposed to the solution, t is the time of exposure, and d is the density. The cup was sealed at a pH of 7.45 and incubated for 7 days at a constant temperature of 37 °C. The pH value of the Kokubo SBF with chemical composition presented in Ref. [24] was measured every 24 h, and the average pH of the SBF was recorded. Kokubo SBF was not replaced or replenished for the time the specimens were immersed, and any gas bubble evolved was observed and noted. The hydrogen evolution rate was assessed during the soaking for 14 days, while the SBF was replenished every day. The electrochemical characteristics of the uncoated and coated samples were evaluated employing PARSTAT 2273 potentiostat/galvanostat (Princeton Applied Research, Oak Ridge, TN, USA). The 1 cm^2^ surface area specimens were plunged in a 3-electrode cell containing Kokubo solution at 37 °C with a pH-value of 7.4. The cell consists of the specimen as the working electrode, saturated calomel electrode (SCE) as reference electrode and platinum as the counter electrode. The potential range of potentiodynamic polarization was between −250 to +250 mV against the open circuit potential (OCP) using the scan rate of 1 mV/s. Electrochemical impedance spectrometry (EIS) was applied after 30 min of specimen placement in the SBF solution to achieve stability in the potential. According to the ASTM G106 standard at an open circuit potential and in the range of 10^5^ to 10^−2^ Hz, this test was carried out using a sine signal with a potential amplitude of 10 mV.

### 2.4. In Vitro Antibacterial Activity

*Escherichia coli (E. coli*) ATCC 25922*,* as a Gram-negative, and *Staphylococcus aureus (S. aureus)* ATCC 25923, as Gram-positive bacteria strains were utilized to evaluate the antibacterial activity of PCL/CS/ZnO coated samples. A 100 μL (10^6^ bacteria) of the bacterial solution was distributed homogeneously on Mueller-Hinton agar, and the PCL/CS/ZnO coated discs with a diameter of 6 mm and placed on the agar for 24 h at 37 °C. The sterile swab dipped into the microbial suspension was flushed (pressing swabs to the side of the pipe) to conduct this, and the cultivation areas were in the form of lawns. Later, the antibacterial performance was evaluated by measuring the inhibition zone, which formed around the PCL/CS/ZnO coated sample according to Ref. [25].

### 2.5. Biocompatibility

To evaluate the cytotoxicity of PCL/CS/ZnO coated sample, 3-(4,5- dimethylthiazol-2-yl)-2,5-diphenyl tetrazolium bromide (MTT, Sigma-Aldrich, St. Louis, MO, USA) assay was performed at 24 and 48 h of culture times. In brief, the specimens (5 mg) were exposed to the culture medium and immersed at 37 °C for 3 and 5 days. Then, on the 96-well plates, 10^4^ cells/mL were refined for 24 h; the cell medium was refreshed according to Ref [25]. The cell counting Kit-8 (CCK-8) assay was employed for the quantitatively analyzed proliferation of MG-63 cell on specimens was according to Ref. [26]. Nuclear staining with DAPI (4′, 6-diamidino-2-phenylindole) followed by fluorescence image analysis (blue fluorescence in live cells) was done to study the MG-63 cell line density of the uncoated and PCL/CS/ZnO coated specimens. The MG-63 osteoblastic cell line was seeded on sterilized samples at a concentration of 2 × 10^4^ cells/mL and incubated for 24 h, after which the cell structures were stained with DAPI and then analyzed employing fluorescence images. Alkaline phosphatase (ALP) activity was considered as a quantitative indicator of bone formation ability. According to Ref. [25], the ALP activity assays were performed for different incubation times to evaluate the effect of ZnO incorporation on primary osteogenic differentiation of MG-63 cells. The cultured cells were washed with phosphate buffered saline (PBS) three times, then fixed with 4% paraformaldehyde for 30 min. After washing 3 times with PBS, they were then stained with ALP staining reagents. The stained cells were washed by PBS three times after holding the cells overnight at 4 °C, and then detected by an optical microscope [25].

### 2.6. Statistical Analysis

Data analysis was expressed as mean ± standard deviation. Statistical analysis was performed by a one-way analysis of variance (ANOVA) using GraphPad Prism software (San Diego, CA, USA, V.7.3). The difference between the data was considered statistically significant at *p* < 0.05 (*) and *p* < 0.01 (**).

## 3. Results and Discussion

### 3.1. Microstructure and Composition

**Figure 2:**
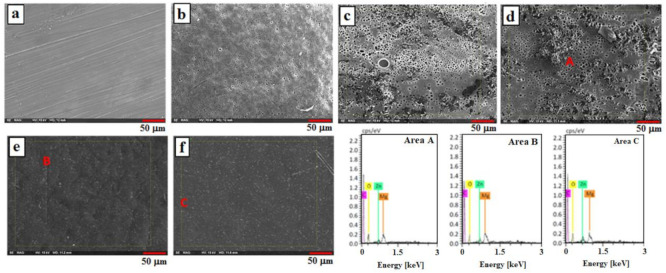
SEM images of (**a**) uncoated, (**b**) PCL/CS, (**c**,**d**) PCL/CS/2ZnO, (**e**) PCL/CS/4ZnO, and (**f**) PCL/CS/6ZnO composite-coated Mg and EDX analysis of Area A, B, and C.

Figure 2 depicts the SEM micrographs of Mg substrate, PCL, PCL/CS, and PCL/CS coating containing ZnO NPs. The presence of some scratches on the mechanically polished uncoated Mg is obvious (Figure 2a). As can be seen, the PCL/CS, and PCL/CS/2ZnO NPs coating layers presented a porous structure with fewer cracks. These pores are generated due to the evaporation of organic solvent during the drying process, as shown in Figure 2b–d. The poor anticorrosion property might be related to these porosities because they could serve as solution channels for further penetration, which cause the underlayer to experience a severe corrosive attack [26]. Although the ZnO NPs were incorporated into the matrix, the pore walls of the polymer were perfectly smooth, and ZnO NPs were seen on the surface of the polymer pore walls. The micro-cracks and micro-pores were not observed on the surface, suggesting that the ZnO NPs were effectively embedded inside the polymer coating. The embedding of ZnO NPs into pores created a reliable and robust composite coating compared to the PCL/CS coating that displayed higher porosity, as shown in Figure 2e,f. The risk of corrosion of the sample may be reduced by the high uniformity of the PCL/CS/2ZnO coating. However, with further addition of ZnO NPs (4 and 6 wt.%), the suggested improvement did not materialise due to the tendency of ZnO NPs to agglomerate.

EDS analysis (Area A–C) was carried out on the surface of the specimens to determine the elemental composition of the coating layer. The presence of a peak related to the Mg and polymer layers was observed. Regarding PCL/CS/2ZnO, PCL/CS/4ZnO, and PCL/CS/6ZnO coatings, other than Mg, C, and O elements, Zn was also detected in the EDS analysis. This is corresponding to the fact that ZnO NPs have been embedded into the polymer matrix. No other element other than carbon, oxygen, magnesium, and zinc was detected, which confirmed that the coating is free of impurities.

**Figure 3:**
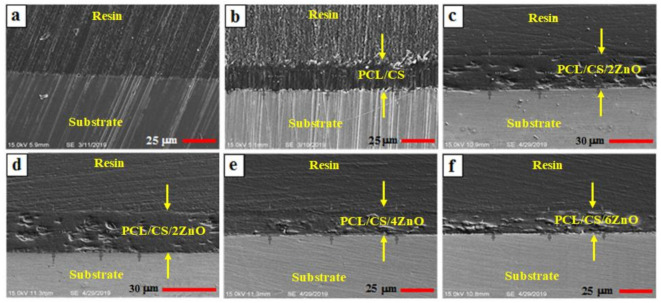
Cross-sectional FESEM micrograph of (**a**) uncoated, (**b**) PCL/CS, (**c**,**d**) PCL/CS/2ZnO, (**e**) PCL/CS/4ZnO, and (**f**) PCL/CS/6ZnO composite-coated Mg.

The SEM image shows that the coating thickness of all specimens is very consistent, which indicates that uniform coatings were successfully deposited on the surface of the Mg substrate. As discussed earlier, this could be due to a viscous solution that produced a uniform and thick layer, which gives better protection to the substrate (Figure 3). Figure 3 presented the cross-section morphology of PCL/CS, and PCL/CS/xZnO-coated Mg specimens. Good integrity can be seen between the PCL/CS and PCL/CS/xZnO coatings and the Mg substrate. The thickness of coatings for the PCL/CS, PCL/CS/2ZnO, PCL/CS/4ZnO, and PCL/CS/6ZnO layers were measured as 20.69 ± 1.87 µm, 21.04 ± 1.05 µm, 19.61 ± 1.52 µm, and 17.47 ± 1.38 µm, respectively. However, ZnO addition from 2 to 6 wt.% has a less significant effect on the thickness of the coating layer. In other words, the addition of ZnO NPs into the polymer matrix resulted in the densification of the coatings with lower porosity, compared to the coatings without ZnO NPs. The composite layer was also found to be completely adhered to the substrate without any cracking or de-bonding at the interfaces, which was a key to enhancing the corrosion protection of the underlayer.

**Figure 4:**
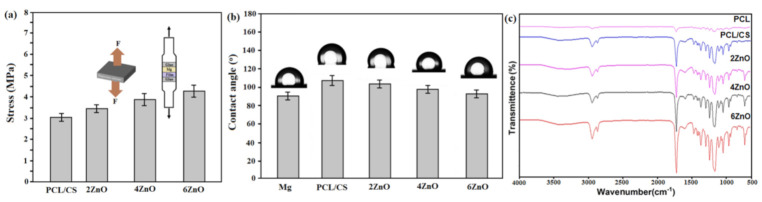
(**a**) Bond strengths, (**b**) Images of water contact angle, and (**c**) FTIR absorption spectra of uncoated Mg, PCL/CS coating, and PCL/CS/ZnO composite-coatings with various amounts of ZnO.

Figure 4a depicts the outcomes of adhesion measurements. The PCL/CS layer on the Mg substrate presented great adhesive strength with the underlayer. It suggests that the bonding strength between the layer and Mg specimen was adequately good to be potentially used in a physiological medium for maintaining a high level of protection from the substrate. The finding depicted that PCL/CS/6ZnO (4.37 MPa), PCL/CS/4ZnO (3.82 MPa), and PCL/CS/2ZnO (3.49 MPa) composite coatings presented higher adhesive strength than that of the PCL/CS without the ZnO (3.12 MPa) coating layer. Furthermore, ZnO good interfacial bonding strength with the polymeric matrix could be described by the hydrogen bonding formed between the =O groups of Zn and the –C sites of the polymer [1,27]. Similar findings suggest that the encapsulation of SiO_2_ nanoparticles may have resulted in an escalation in the bonding strength of multilayer films [28]. The influence of ZnO NPs-incorporated PCL/CS coating layers on the surface properties was studied by measuring the contact angle (CA). Figure 4b demonstrates that PCL/CS coating layers showed a CA of 106.1°, which is consistent with other research [29]. When the weight percent of the loading ZnO NPs increases from 2 to 6 wt.%, the contact angles values gradually declined to 92.6° (PCL/CS/6ZnO), indicating increased roughness and more hydrophilic surface. Based on the CA measurement, it is predictable that due to the incorporation of ZnO NPS, resulting in a modified surface, that affect both coating adhesion and biological activity.

Figure 4c depicts the FTIR spectrum of the PCL/CS/xZnO composite specimen. Generally, the typical PCL peak dominates the FTIR spectrum of the PCL/CS/ZnO composite. An intense peak at 1723 cm^−1^ is seen in the PCL coating, which corresponds to the –C=O stretching in the PCL polymer’s carbonyl ester group. The C–H related to saturated carbon was seen at 2867 and 2944 cm^−1^ [29]. The bands at 1162 cm^–1^ to 1366 cm^–1^ indicate the amine, –CN stretch in chitosan. The band at 1596 cm^–1^ is attributed to the –NH bond in chitosan. The chitosan spectrum shows a peak at 3438 cm^–1^ because of the stretching vibration of hydroxyl and amino groups of chitosan (CS) [30]. The bands at 500–700 cm^−1^ that are related to the stretching of the Zn–O bond are present in the PCL/CS/(2–6 wt.%)ZnO coatings [20].

### 3.2. Morphological Analysis of Corroded Surfaces and Properties

**Figure 5:**
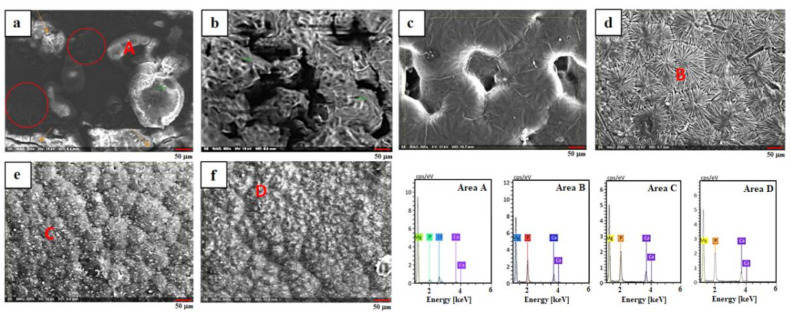
FESEM micrographs of (**a**,**b**) uncoated Mg, (**c**) PCL/CS, (**d**) PCL/CS/2ZnO, (**e**) PCL/CS/4ZnO, and (**f**) PCL/CS/6ZnO composite-coated Mg specimens and EDX analysis of Area A, B, C, and D.

The morphologies and corresponding element contents of the specimens after the 7 days immersion are presented in Figure 5. For the bare Mg (Figure 5a), corrosion occurred and corrosion products could be found on its surface. The specimen with PCL/CS/ZnO coating showed fewer corrosion cracks and more Ca-P products than the bare and PCL/CS coated specimens (Figure 5b,c), indicating good corrosion performance of the PCL/CS/ZnO film. The Zn–OH groups were generated, and then these groups would induced Ca^2+^ and PO_4_^−3^ ions, which were available on the surface in the solution. Corrosion products with flake morphology for the coating with 2 wt.% ZnO and cauliflower morphology for coating with 4 and 6 wt.% ZnO could be clearly seen on the surface of the specimen (Figure 5d–f). The formation of the corrosion products with flake morphology could act as a second barrier and further protect the solution penetration into the substrate, thus improving the corrosion resistance [27]. Similarly, it was reported [29] that the Zn ion released from PCL/ZnO composite layer in the body could promote bone formation.

Based on the EDS analysis (Area A–D), it could be determined that the Ca/P ratios for the bare, PCL/CS, PCL/CS/4ZnO, and PCL/CS/6ZnO-coated Mg were 1.19, 1.27, 1.42, and 1.46, respectively, which were lower than that of standard hydroxyapatite (Ca/P = 1.67). The information revealed that calcium phosphate layers with low calcium content emerged on the surface of the specimens when they were immersed in the corrosive medium. In general, owing to the corrosion of specimens by the corrosive solution, Ca^2+^ ions in the hydroxyapatite structure could be substituted by the released Mg^2+^ ions [31]. Therefore, it could be determined that the samples modified by the PCL/CS/ZnO film may improve hydroxyapatite formation, which could supply further anti-corrosive protection for Mg substrates.

**Figure 6:**
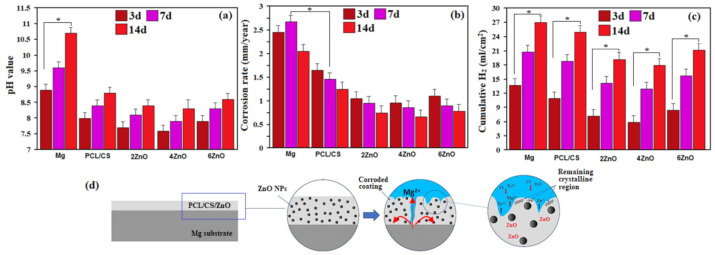
(**a**) pH value, (**b**) corrosion rate, (**c**) hydrogen evolution for uncoated (Mg) and PCL/CS coating, and PCL/CS/ZnO composite-coatings with various amounts of ZnO and (**d**) schematic of the corrosion mechanism of PCL/CS/ZnO composite-coated Mg specimens after soaking in SBF (* *p* < 0.05).

The initial mass and pH value of each sample were measured and recorded before immersion. The changes in pH value resulted in the corrosion of the specimen produced corrosion product, increasing the basicity of the SBF solution. As shown in Figure 6a, the pH value of the specimens showed an increasing trend throughout the seven days of immersion. The solution with bare Mg sample showed the highest pH value among all the specimens, indicating that the Mg has the highest corrosion activity when immersed in SBF solution compared to the coated specimens. The aggressive ions could quickly attack the uncoated Mg in the SBF solution as it is a reactive metal, resulting in pitting corrosion on the Mg surface. Uniform corrosion can also occur, depleting Mg slowly, causing thinning of Mg, and dissolving all the Mg samples. This is indicated by the considerable loss of mass of Mg after seven days of immersion. The coated specimens had a significantly lower pH (8.52) compared to the uncoated Mg (10.7). The PCL layer, which poses hydrophobic property, had slowed down the corrosion process. This behavior originated from the acidic products of polymer degradation [26]. PCL/CS containing ZnO NPs presented a low pH trend line among the specimens related to the lower corrosive products being released into the SBF solution. Furthermore, by increasing the concentration of ZnO, the pH value became higher.

The corrosion rates calculated based on the mass loss of uncoated and coated specimens are demonstrated in Figure 6b. All coated specimens showed significantly lower mass loss than the bare sample, which shows the efficiency of PCL/CS and PCL/CS/ZnO coatings in decreasing the corrosion rate of the substrates. Similarly, mass loss of specimens increases as the ZnO concentration increases from 4 wt.% to 6 wt.%. It has been mentioned that metal oxide contributes towards the PCL matrix hydrolysis, which can increase the corrosion activity of the PCL [27,29]. PCL/CS had a higher corrosion rate (1.25 mm/year) than uncoated (2.1 mm/year) due to the hydrophilic property of the polymer layer. Subsequently, the average in-vitro corrosion rate of PCL/CS/4ZnO coatings is about (0.73 mm/year) in SBF solution at 37 °C. Therefore, it can be concluded that the layer of the PCL/CS/ZnO coating had the highest protection in the Mg substrate.

Figure 6c shows the profiles of hydrogen evolution of the PCL/CS/xZnO coatings. It can be concluded that the released hydrogen volume had the following order: PCL/CS/ZnO < PCL/CS < Mg, throughout the entire test period. Uncoated pure Mg substrate displayed a rapid evolution of hydrogen. At the same time, PCL/CS coated specimens possessed a nearly constant hydrogen evolution rate marginally reduced compared to PCL/CS/(4–6)ZnO. On the contrary, PCL/CS/4ZnO specimens displayed very low hydrogen evolution, showing a significantly lower degradation rate owing to a composite coating compared to the uncoated substrate and polymeric coatings alone. The addition of ZnO nanoparticles into the polymer-based matrix decreased the hydrogen evolution rate, which could be caused by decreasing the polymer layer number of micro-pores. However, there was no significant difference between the hydrogen evolution rate of PCL/CS and the PCL/CS/ZnO coating (*p* > 0.05). Overall, for neat Mg, rapid corrosion rate, intense generation of hydrogen, and local pH escalation occurred throughout the soaking period. However, for the PCL/CS film with ZnO, which could be owing to the encapsulation of ZnO, the degradation rate, hydrogen release, and pH escalation is greatly inhibited, which can boost the compactness of the coating, resulting in greater composite coating corrosion protection.

Figure 6d illustrates the schematic degradation mechanism of the PCL/CS/ZnO composite coatings on the Mg substrate in SBF solution. When the coated specimens were placed in the solution, the electrolyte reached the interface of the porous and barrier layers because the corrosive solution entered the coating layer via the outer porous layer. Because of the composite coating layer dense structure, the barrier layer electrolyte diffusion is a slow reaction. The incorporation of 2 wt.% ZnO NPs will increase the coating effectiveness because of a rise in the barrier layer thickness and the barrier layer effectiveness due to a decreased quantity of the pores of the surface [2,29]. This could be attributed to the fact that the coating with the highest density and lowest porosity could be attained in this concentration of ZnO NPs, consistent with FESEM observations and polarization test results. Increasing the ZnO NPs loading to 6 wt.% resulted in a decreased protective level of the coating layer because of reduced barrier layer effectiveness as a result of ZnO NPs agglomerations. In particular, the amount of porosity diminished by encapsulation of ZnO NPs into the porosity at which aggressive solution infiltration routes are formed is dramatically diminished, contributing to the relatively lower rate of corrosion of the entire coating, supplying the Mg with a high level of protection for a prolonged duration.

### 3.3. Electrochemical Behavior

**Figure 7:**
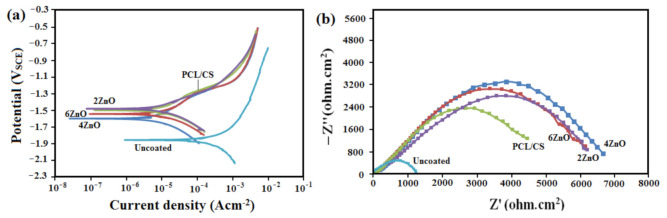
(**a**) Electrochemical anodic potentiodynamic polarization curves and (**b**) Nyquist curves of uncoated and PCL/CS/ZnO composite-coated Mg specimens.

Potentiodynamic polarization is an effective test for evaluating the corrosion rate of the materials. The curves obtained from this test for the bare and coated Mg specimens are depicted in Figure 7a. The attained curves for PCL/CS (−1498 ± 14 mV_SCE_), and PCL/CS/2ZnO coated (−1492 ± 12 mV_SCE_) specimens revealed that the corrosion resistance was higher than that of the uncoated Mg specimen (−1887 ± 18 mV_SCE_), which shows higher corrosion resistance of the coated specimens compared to the bare sample. The corrosion potentials (E_corr_) of the coated samples shifted to a more positive value in the range of (−1492 ± 12 to −1616 ± 14 mV_SCE_), indicating the specimens thermodynamic stability state compared to the uncoated sample. The rise in thermodynamic stability of the PCL/CS/ZnO coated specimen could be due to the ceramic nature of the deposited ZnO layer with low concentration and its high chemical stability in the physiological environment as well as the formation of the polymer layer of Mg surface, which acts as a barrier layer to prevent solution penetration. Our findings has a good agreement with Refs. [13,31]. The specimens degradation rate is generally attained by measuring the corrosion current density (*i*_corr_), in which the lower *i*_corr_ shows the lower corrosion rate. The *i*_corr_ value for uncoated Mg was the highest among all specimens, revealing that it is susceptible to easy attack by corrosive media. The *i*_corr_ value decreased significantly for the composite-coated substrate, indicating the coated specimens higher chemical stability in corrosive media. The results showed that all coated specimens had higher corrosion resistance than the bare Mg specimen, indicating composite coatings effectiveness in decreasing the corrosion rate of the substrates. The incorporation of 2 wt.% of ZnO NPs to polymeric matrix increased the corrosion resistance, possibly resulting in a decrease in the number of micro-pores in discharging channels. The addition of metal oxide (4 and 6 wt.% ZnO) will increase the polymer matrix hydrolysis, thus reducing the thickness of the substrate.

The corrosion resistance of the specimens was determined through electrochemical impedance spectroscopy (EIS), as shown in Figure 7b. The EIS results interpretation is made by studying the correlation between impedance data and the equivalent circuit, where R_S_, Q, and Qb are the solution resistance, constant phase element, and double layer capacitance, respectively. WE showed the working electrode (sample), and Rb showed charge transfer resistance associated with micro-galvanic events paralleled to the electrical double layer at the electrolyte solution interface and the Mg matrix (Qb), as shown in Figure S3. In this regard, the capacitive loop diameter shows the resistance of charge transfer (Rb), where the larger the diameter of the semicircle diameter, the higher the resistance, and hence, the lower the corrosion rate. The Rb of PCL/CS/2ZnO is (6567 ± 22 Ω·cm^2^), which is significantly higher than the Rb of uncoated Mg (943 ± 13 Ω·cm^2^), which implies significantly higher corrosion resistance than uncoated pure Mg substrate. All coated specimens have a larger capacitive loop diameter, which proved that the PCL/CS-based film could enhance Mg initial corrosion resistance. Among the coated samples, the one with 4 wt.% ZnO NPs displayed a larger loop (6732 ± 26 Ω·cm^2^), showing the highest resistance of corrosion among all specimens. The results are consistent with the polarization test results. By increasing the ZnO concentration to 6 wt.%, the loop diameter decreases, which indicates the coating corrodes faster and possesses lower R_b_. This is due to hydrolysis and degradation of the polymer matrix, leading to more penetration of aggressive ions in SBF solution into the substrate [6] and reducing the coating layer level of protection.

### 3.4. Cellular Response

**Figure 8:**
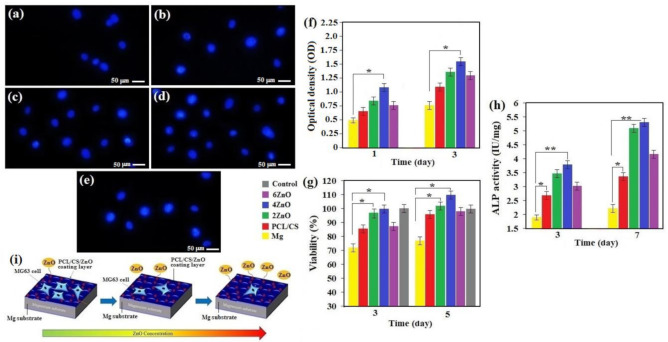
(**a**) DAPI staining of MG-63 cells cultured on a sample (**a**) uncoated, (**b**) PCL/CS, (**c**) PCL/CS/2ZnO, (**d**) PCL/CS/4ZnO, (**e**) PCL/CS/6ZnO and, (**f**) CCK-8 assay; and (**g**) cell viability and (**h**) ALP activity of MG-63 cells cultured for various times on uncoated and coated samples and (**i**) schematic demonstration of the interactions between the MG-63 osteoblast cells and the PCL/CS/ZnO composite coated sample (* *p* < 0.05 and ** *p* < 0.01).

To evaluate the biological response on the surface of bare and coated Mg, osteoblast cells were incubated for 24 h, inspecting cell attachment via a fluorescence image. In Figure 8a–e, DAPI staining of cell nuclei on bare and PCL/CS/ZnO coated Mg revealed a limited number of cell nuclei attached to the surface of the uncoated specimen after 24 h of culture. The results revealed that the uncoated Mg specimen surface could not provide a good environment for the cells to survive because cracks and hydrogen release resulted from uncontrolled corrosion [17]. Alternatively, PCL/CS and PCL/CS/ZnO coatings Figure 8b–e revealed drastically improved cell adhesion and density, while the amount of the cells remarkably diminished on the PCL/CS/ZnO coating containing 6 wt.% ZnO, suggesting that extra Zn ions caused an adverse influence on the cells [21]. Ultimately, the PCL/CS/ZnO coatings demonstrated the greatest potential to stimulate cell proliferation, and the ZnO particles have a great capacity to compensate low bioactivity of PCL film by generating Zn^2+^. In this regard, it was reported that the hybrid composite incorporated with ZnO is able to promote the formation of new bone tissue along with new blood vessels; such properties are called osteogenic effect and angiogenic effect, respectively [27,32]. The finding shows that ZnO can accelerate the biological process that can lead to organ repair function through its ability to stimulate new tissue formation. In general, the optical density (OD) value was proportional to the number of viable cells. The optical densities in the extracts of PCL/CS/ZnO-coated Mg were greater than that in the uncoated sample, and the cell activity escalated as the culture period extended (Figure 8f). Moreover, during the cultivation period, a greater optical density was found in the PCL/CS loaded with low ZnO extract than that of the coating loaded with higher ZnO extract. Figure 8g demonstrates the results of the viability of the cells co-cultured with extracts of all pure Mg and coating samples. Because of the uncoated Mg substrate’s initial rapid corrosion, the generated hydrogen and cell viability level are very low. By contrast, cell viability increased significantly for PCL/CS and PCL/CS/ZnO coating with a low amount of ZnO. It is recognized that cells are susceptible to changes in the micro surrounding environment, like the sharp changes in the Mg^2+^ concentration and pH value [33]. The sharp rise in the concentration of Mg^2+^ and pH value released by the substrates’ corrosion and the PCL and PCL/CS coatings with a high amount of ZnO coating specimens may reduce cell viability. It can be seen that with increasing culture time, none of the specimens displayed cytotoxicity, and the cell survival rate remained higher than 80%.

After incubation of 3 and 5 days, it was observed that all coating extracts improved cell viability, particularly after 5 days of incubation; the PCL/CS and PCL/CS with low ZnO content presented a higher cell survival rate of 110%. This may be because of the substrate’s higher protection performance, which prevents the excess ions from entering the culture medium. Hence, it can be determined that composite coatings are a more appropriate option to improve the proliferation and growth of osteoblasts [33]. It has been reported that Zn-encapsulated montmorillonite coatings enhanced cellular compatibility may be attributed to the beneficial effect of Zn^2+^ ions, which help the activity and proliferation of bone cells [7]. The incorporation of Zn into a composite coating may help the spread and adhesion of cells. Surprisingly, a significant difference was observed in cell viability between the PCL/CS/ZnO coatings and the uncoated sample (*p* < 0.05). In-vivo evaluation of biocompatibility of PCL/CS coatings containing ZnO or cytotoxicity to cells, tissues, and organs is one limitation of this research.

ALP activity is a quantitative indication for osteogenesis performance. In the ALP study, MG-63 cell response to PCL/CS/ZnO coating was regarded as using ALP assay to evaluate its effect on osteogenesis (Figure 8h). An increase in ALP activity was observed by escalating the time of incubation. After 3 and 7 days of culture, ALP activity of the PCL/CS and PCL/CS with low ZnO amount displayed a substantial improvement compared to PCL and PCL/CS with high ZnO amount. The findings pointed out that PCL/CS with low ZnO content might assist in cell differentiation, implying that ZnO-encapsulated PCL/CS film positively affected the ALP activity of cells attached to the coating film [27]. The ALP expression of the substrates and the PCL/CS/ZnO coatings displays a consistent result with the MTT assay.

It is worth mentioning that PCL/CS, a biodegradable material, can work as a physical barrier layer and provide an appropriate condition for cell adhesion and proliferation on the Mg substrate (Figure 8i). The combination of PCL with CS and a small amount of ZnO can improve the coating’s performance on biodegradable Mg. Regarding surface chemistry, the presence of CS with hydrophilic nature properties displayed higher surface chemical property towards osteoblast cells, which would play a significant part in enhancing cytocompatibility [21]. Because of the excellent protection of PCL/CS and PCL/CS/ZnO coating, Mg^2+^ and OH^−^ release became significantly slower in a more controlled manner, resulting in a near-physiological condition with acceptable Mg^2+^ concentration, pH, and osmolality for osteoblast cells function, viability, and proliferation [15,29,32]. Throughout the entire assay, PCL/CS/4ZnO displayed outstanding cell viability compared to the other groups, including the PCL/CS/xZnO, indicating that the composite coating on the Mg specimen has no cytotoxicity effect on osteoblast cells, but has benefits for cell growth.

### 3.5. Antibacterial Studies

**Figure 9:**
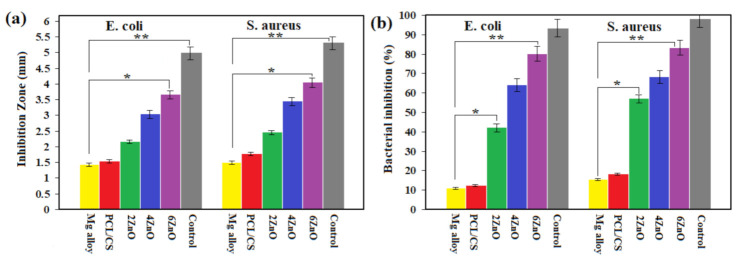
(**a**) Values of growth inhibition zones and (**b**) and percentage of bacterial inhibition against *S. aureus* and *E. coli* bacteria of uncoated, and PCL/CS/6ZnO and (Control): Gentamicin (* *p* < 0.05 and ** *p* < 0.01).

Figure S4 depicts the inhibition zones for the uncoated PCL/CS, and PCL/CS/ZnO coated specimens against the two tested bacteria—*E. coli* and *S. auras*. The inhibition zones diameters are tabulated in Figure 9a. No inhibition zone was seen around the uncoated specimen and PCL/CS-coated samples for both organisms. For PCL/CS/ZnO, the inhibition zone against the *E. coli* and *S. aureus* bacteria was about 5.2 mm, indicating the antibacterial properties of ZnO NPs. Besides, there was no remarkable change in the samples with 2 wt.% and 4 wt.% of ZnO; however, the sample having 6 wt.% of ZnO displayed an improved antibacterial property. Likewise, Figure 9b shows that the bacterial elimination percentage (*E. coli* and *S. aureus*) for PCL/CS coatings with various content of ZnO NPs (2–6 wt.%) is significantly higher than uncoated Mg and PCL/CS coating. In this regard, PCL/CS/6ZnO presented a significantly higher rate of bacterial elimination than other PCL/CS-based coatings, representing the synergetic influence of CS and ZnO NPs on bacterial elimination of the coating layer. In this context, significant differences between the antibacterial activity of the uncoated and PCL/CS/ZnO coatings were observed (*p* < 0.05). The enhanced bactericidal properties of the PCL/CS/ZnO coating could be increased with increased ZnO concentration and the quantity of ZnO NPs per volume unit raised, increasing the surface area generation of hydrogen peroxide (H_2_O_2_) [21]. In this context, Perelshtein et al. [34] implied that although the higher concentration of ZnO added to CS increases its antibacterial effects, the combined effects of CS and ZnO improved the antibacterial activity of the two applied organisms. Chitosan encapsulated with ZnO [18] has shown a higher inhibition zone against Gram-negative bacteria such as *E. coli* compared to ZnO alone, which means the addition of CS enhanced the antibacterial activity of ZnO. A similar study conducted by Yusof et al. [35] also suggested that the combination of ZnO and CS can control Gram-negative and Gram-positive bacteria growth. The outstanding antimicrobial activity of the PCL/CS/ZnO composite coating was due to the synergistic antimicrobial properties of CS and ZnO NPs. This result in the production of reactive oxygen species (ROS) like H_2_O_2_, where CS electrostatically binds to the bacterial cell wall while ZnO NPs interact with protein membrane, resulting in the intracellular released material and perforated cell membrane [36,37,38,39,40,41,42,43,44,45,46,47,48,49,50].

## 4. Conclusions

The PCL/CS composite coating with various ZnO NPs amounts was fabricated on the Mg surface to improve corrosion resistance and bacteriostatic effect. In this context, PCL/CS can reduce the Mg in vitro degradation rate, whereby ZnO NPs releasing from PCL/CS coating leads to enhancement of antibacterial activity. The PCL and PCL/CS coatings have a porous structure with thickness of about 50 μm; after ZnO nanoparticles were embedded in the coating, the compactness and uniformity of the coatings was enhanced. The composite coating significantly reduced Mg substrate corrosion in SBF solution and cell culture medium, meeting clinical requirements. Furthermore, the PCL/CS and PCL/CS layer deposition with low ZnO NPs content generated a more favorable interface and microenvironment for osteoblastic cells, displaying outstanding cytocompatibility and improved osteogenic differentiation. At the same time, further incorporation of ZnO NPs has a reverse effect. Electrochemical and immersion tests, including mass loss and H_2_ evolution, exhibit that the corrosion resistance of PCL/CS and PCL/CS/ZnO coatings were noticeably higher than that of the uncoated Mg. It also reveals improved antibacterial performance against *E. coli* and *S. aureus* bacteria, where antibacterial effects increased with increased ZnO concentration. The results are of significance in PCL/CS/ZnO layer design as coatings, to improve degradation resistance and enhance antibacterial performance to inhibit orthopedic infections.

## Data Availability

All the relevant data used in the study have been provided in the form of figures and tables in the published article, and all data provided in the present manuscript are available to whom it may concern.

## Supplementary Materials

The following are available online at https://www.mdpi.com/article/10.3390/ma14081930/s1, Figure S1, Figure S2, Figure S3 and Figure S4.
