# Supplementary material for: Improved Bacteriostatic and Anticorrosion Effects of Polycaprolactone/Chitosan Coated Magnesium via Incorporation of Zinc Oxide
Source: Materials (Basel). 2021 Apr 12;14(8):1930. doi: 10.3390/ma14081930 (PMC8070643; doi:10.3390/ma14081930)
Supplement: Supplementary file 1 [file materials-14-01930-s001.pdf]

materials

Article

Supplementary Material

Improved Bacteriostatic and Anticorrosion Effects of Polycaprolactone/Chitosan Coated Magnesium via Incorporation of Zinc Oxide

Hamid Reza Bakhsheshi-Rad [1,2,*], Esah Hamzah [2,*], Wong See Ying [2], Mahmood Razzaghi [1], Safian Sharif [2], Ahmad Fauzi Ismail [3] and Filippo Berto [4,*]

[1] Advanced Materials Research Center, Department of Materials Engineering, Najafabad Branch, Islamic Azad University, Najafabad, Iran; mahmood.razzaghi@gmail.com
[2] Faculty of Engineering, Universiti Teknologi Malaysia, Johor Bahru, Johor 81310, Malaysia; seeying1202@gmail.com (W.S.Y.); safian@utm.my (S.S.)
[3] Advanced Membrane Technology Research Center (AMTEC), Universiti Teknologi Malaysia, Johor Bahru, Johor 81310, Malaysia; afauzi@utm.my
[4] Department of Mechanical and Industrial Engineering, Norwegian University of Science and Technology, 7491 Trondheim, Norway
* Correspondence: esah@mail.fkm.utm.my (E.H.); rezabakhsheshi@gmail.com (H.R.B.-R.); filippo.berto@ntnu.no (F.B.)

Citation: Bakhsheshi-Rad, H.R. Hamzah, E.; Ying, W.S.; Razzaghi, M. Sharif, S.; Ismail, A.F.; Berto, F. Improved Bacteriostatic and Anticorrosion Effects of Polycaprolactone/Chitosan Coated Magnesium via Incorporation of Zinc Oxide. *Materials* **2021**, *14*, x. https://doi.org/10.3390/xxxxx

Academic Editor: Giuseppina Sandri

Received: 11 February 2021
Accepted: 5 April 2021
Published:

Publisher's Note: MDPI stays neutral with regard to jurisdictional claims in published maps and institutional affiliations.

Copyright: © 2021 by the authors. Licensee MDPI, Basel, Switzerland. This article is an open access article distributed under the terms and conditions of the Creative Commons Attribution (CC BY) license (http://creativecommons.org/licenses/by/4.0/).

1. Casting Procedure

For preparation of as-cast Mg substrates, pure Mg ingot (99.9% Mg) was used as starting materials. The materials were melted under argon gas in a mild steel crucible at a temperature of 740 °C for 45 min holding time. Following melting, the molten metal was poured into a pre-heated mild steel mold to attain ingots. The surface morphology of the uncoated specimens, as cast magnesium substrate was observed using optical microscope is shown in Figure S1. It shows the presence of grain boundaries.

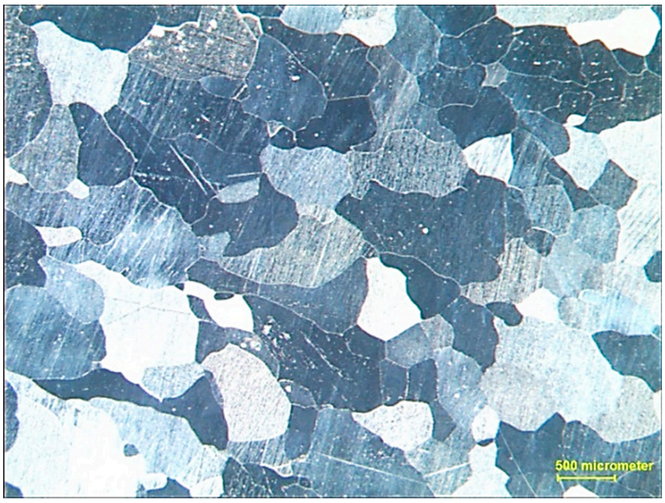

Figure S1. Optical Micrograph of as-cast Mg.

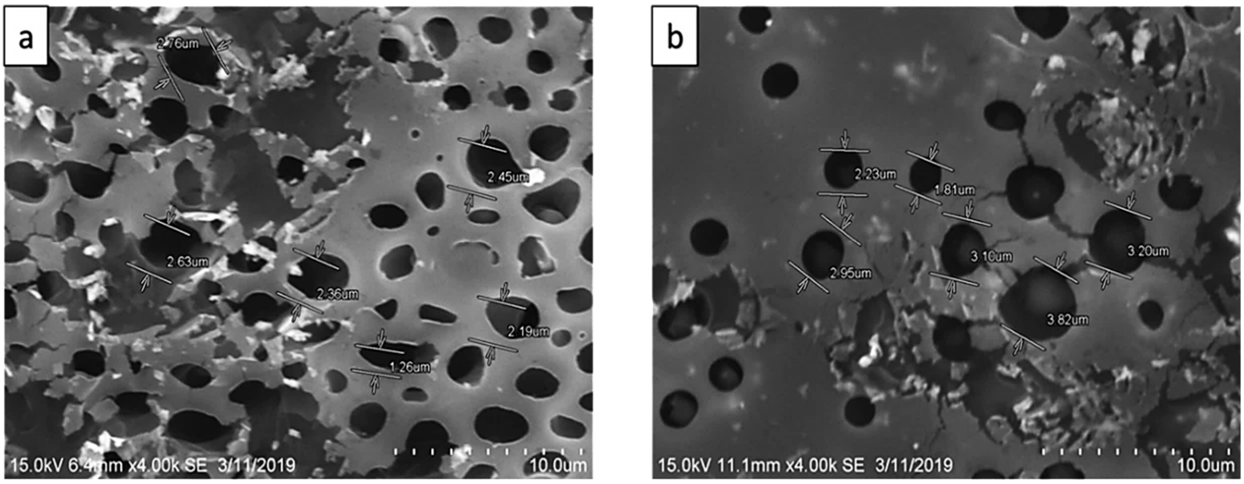

Figure S2. SEM images of (**a**) Mg coated with PCL/CS, (**b**) Mg coated with PCL/CS/ZnO.

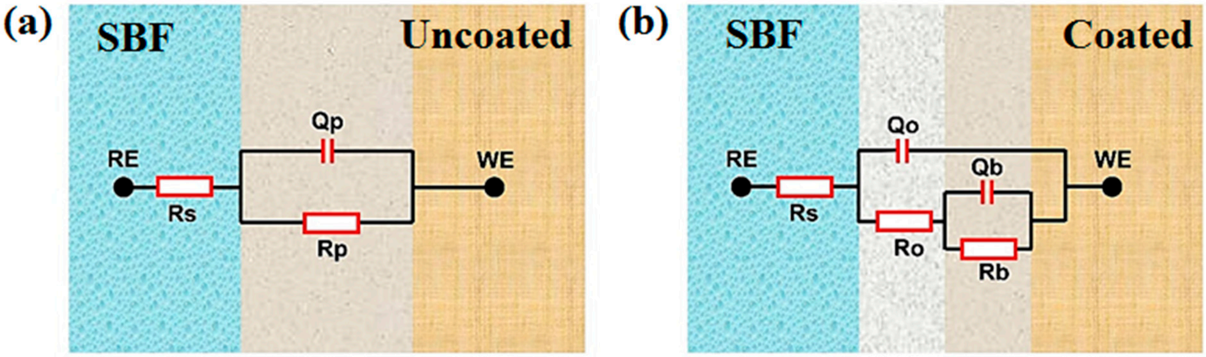

Figure S3. Equivalent circuits used to fit the EIS diagrams for the (**a**) uncoated and (**b**) PCL/CS/ZnO coated samples in SBF solution in ambient conditions.

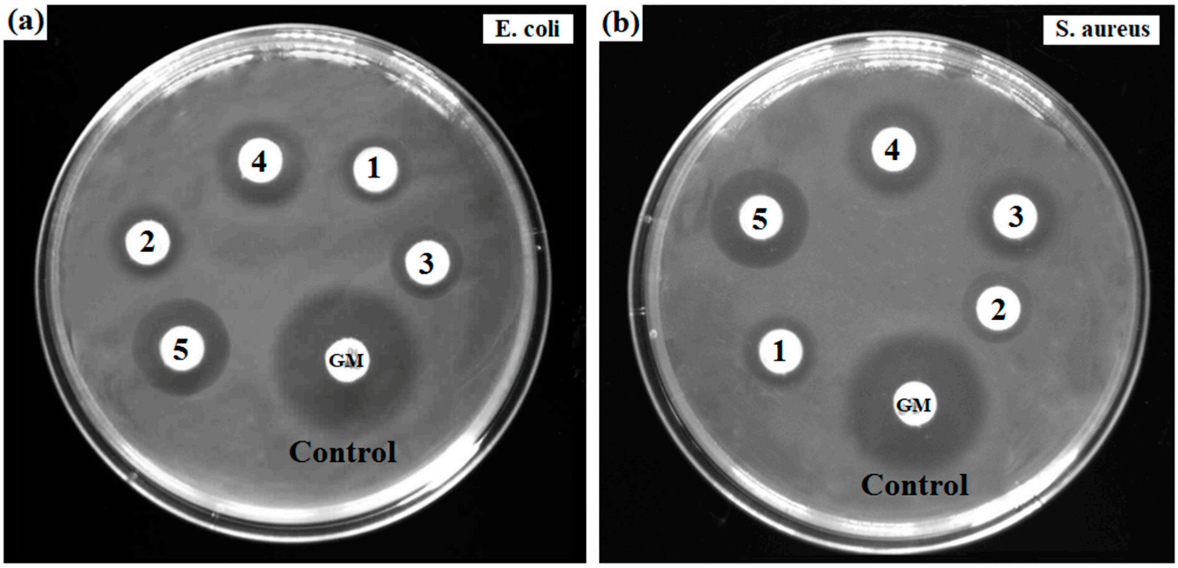

Figure S4. Images of inhibition zones of the uncoated, PCL/CS, and PCL/CS/ZnO coated samples after 24 h against (**a**) Gram-negative (*E. coli*) and (**b**) Gram-positive (*S. aureus*). Note: (1): uncoated; (2): PCL/CS; (3): PCL/CS/2ZnO; (4): PCL/CS/4ZnO; (5): PCL/CS/6ZnO and (Control): Gentamicin.
